# Respectability and boundary making on a superdiverse housing estate: The cross‐racial deployment of intra‐ethnic stereotypes

**DOI:** 10.1111/1468-4446.12922

**Published:** 2022-01-19

**Authors:** James Rosbrook‐Thompson, Gary Armstrong

**Affiliations:** ^1^ Department of Sociology City, University of London London UK

**Keywords:** conviviality, ethnicity, race, respectability, superdiversity, symbolic boundaries

## Abstract

This article examines how white British residents of a superdiverse London housing estate learn about—and subsequently deploy—the intra‐ethnic stereotypes used by their British Pakistani and British Bangladeshi neighbours/flatmates. Building on recent attempts to bring together conviviality and boundary making, along with insights into intra‐ethnic othering, we show how, for white British residents, these stereotypes offered the chance to add detail and authenticity to judgements about the “unrespectable” behaviour of British Asian residents and/or visitors. Ultimately, however, white British residents' inappropriate and/or imprecise deployment of these stereotypes in relation to British Bangladeshis and British Pakistanis led to the misidentification of low‐status people and the unfair extension of discrimination faced by low‐status individuals and families. Furthermore, the combination of clumsy application and the positioning of “respectable” British Bangladeshis and British Pakistanis as purveyors of “insider knowledge” about intra‐ethnic stereotypes led to the reinscribing of boundaries between racial groups. We conclude that studying the cross‐racial use of intra‐ethnic stereotypes allows for a subtler appreciation of the complex dynamics of inclusion and exclusion in superdiverse areas.

## 1 INTRODUCTION

Judgments regarding respectability are often used to draw and maintain symbolic boundaries between groups (Elias & Scotson, 1994; Skeggs, 1997). In superdiverse urban settings these judgments correspond with various categories including “race,” ethnicity, a more general “foreignness,” together with “established‐outsider” and “established‐newcomer” distinctions (Albeda et al., 2018; Wessendorf, 2020).

In many ways boundary‐making dynamics underline the ability of conviviality or *convivencia* (Gilroy, 2004) to capture both the inclusionary and exclusionary realities of urban life, especially where instances of convivial practice are foregrounded (Wise & Noble, 2016). In the case of judgments about respectability, processes of othering and attempts to cross the boundaries created and reproduced by these processes involve convivial practices that vary in their duration and intensity. These range from relatively thin and effortless interactions, or easy conviviality (Wessendorf, 2020), to more strenuous, concerted attempts to negotiate concrete differences between ethnic groups, or convivial labor (Wise, 2016).

A focus on both inclusion and exclusion ensures that judgments are set in the context of structures of power and inequality with, for example, socio‐economic disadvantage playing a significant role in determining the contours of symbolic boundaries (Wessendorf, 2020). More broadly, this focus reflects a critical stance in relation to the realities of superdiversity, avoiding the pitfalls of an unreflective and celebratory diversity narrative (Alexander & Nayak, 2016; Back, 2015; Rosbrook‐Thompson, 2018) by remaining attentive to what Back (2009) has called the “metropolitan paradox”—that is, the co‐existence of racism *and* conviviality within everyday urban settings. This paradox has also been explored in a suburban location, where notions of whiteness and everyday constructions of home shape the dynamic between inclusion and exclusion (Tyler, 2015, 2017, 2020).

Though superdiversity concerns the proliferation and accentuation of intra‐ethnic differences (Vertovec, 2007), little is known about the processes of othering and boundary making which occur within ethnic groups in superdiverse areas. Where intra‐ethnic boundaries upheld by judgments around respectability have been explored, such as in Charsley and Bolognani's (2017) study of the “freshie” stereotype applied to Pakistani newcomers by British Pakistanis, the focus is solely on intra‐ethnic interactions and dynamics. Indeed, we know almost nothing about how such intra‐ethnic boundary making (and maintenance) is drawn upon in engagement across racial lines and the convivial labor this may entail.

In what follows we use the literature on superdiversity, conviviality and symbolic boundaries to examine how intra‐ethnic distinctions feature in the inter‐racial exchanges which take place on a superdiverse London housing estate.^1^ The focus is on white British residents of the estate who did not grow up in the area and their exchanges with neighbors and flatmates who were from British Asian backgrounds. More specifically, we explore how white British residents use knowledge of intra‐ethnic boundaries gleaned from inter‐racial exchanges to formulate judgments about (un)respectable behavior. Ostensibly these judgments resulted from well‐intentioned attempts to understand the conduct of British Bangladeshi and British Pakistani residents and visitors in ways that were more granular and culturally aware, through inter‐racial exchanges that were entered into proudly and reflected a stated commitment to perform the convivial labor necessary to live on a superdiverse estate. For example: Why were the British Bangladeshi family upstairs routinely eating so late (and noisily) at night? And how could the issue be addressed in a way that avoided racist stereotyping by showing some understanding of “authentic” intra‐ethnic boundaries?

The paper supplements our knowledge of intra‐ethnic boundaries by showing how these boundaries are deployed in the context of cross‐racial judgments about respectability. In analyzing such judgments and the inter‐racial exchanges through which they are formulated, we arrive at a subtler appreciation of how convivial labor is implicated in the dynamics of inclusion *and* exclusion in superdiverse settings. The paper also sheds light on how everyday constructions of home—in an immediate, material sense (as residence) and more widely as a superdiverse locale—feature in urban conviviality's play of inclusion and exclusion.

In the following section we discuss existing research on superdiversity, symbolic boundaries, conviviality and convivial labor. We then describe the context of the ethnographic research together with the mechanics of the fieldwork itself. In presenting the findings of the research we detail two respective intra‐ethnic boundaries that were evident on the estate, then explore how the deployment of these boundaries by white British residents involved processes of inclusion and exclusion.

## 2 SUPERDIVERSITY, CONVIVIALITY, AND BOUNDARY MAKING

Back (2009) has noted the propensity of conviviality and racism to co‐exist within everyday multicultural settings—what he calls the “metropolitan paradox.” The paradox is poignantly conveyed in Back and Sinha's (2018) ethnography conducted collaboratively with 30 migrants in London, where convivial inter‐ethnic exchanges are woven together with recollections of racism and xenophobia. Identifying superdiversity and/or conviviality with only the frictionless elements of urban life represents an unreflective “celebratory diversity narrative” (Back, 2015) which overlooks the realities of racism (Alexander & Nayak, 2016) and corresponds with a failure to interrogate how ostensibly positive, progressive attitudes to diversity may lack substance (Ahmed, 2010). Indeed, in outlining the concept of conviviality Gilroy (2004) pointed to its “negative dialectics,” while Wise and Noble (2016) underline the capacity of conviviality—and, more specifically, the Spanish notion of *convivencia*—to encompass “happy togetherness” *and* negotiation, friction and occasional conflict.

Researchers have responded by charting complex patterns of inter‐racial and inter‐ethnic engagement in urban space and foregrounding the situated practices through which differences are identified and negotiated. In Hackney, East London, Wessendorf (2014a, 2014b) describes varying levels of engagement with the area's “multiplex differences” across public, private, and parochial spheres. Whereas in the public realm there is a “civility towards diversity” or “easy conviviality,” the parochial realm (which includes places such as schools and sports clubs) demands more concrete acknowledgment of, and interaction across, categorical differences, or as Noble (2009, p. 53) described it, “the labour of intercultural community.” In a similar vein, Wise (2009) has written of the “convivial labour” involved in the framing of humor in multi‐ethnic workplaces. One of the forms this labor takes is “frame negotiation,” which Wise (2009, p. 482) defines as “the enacted, negotiated, practiced and cumulative labour that goes into provisionally successful situations of lived difference.”

This relationship between judgments of acceptability and the framing of humor in the multi‐ethnic workplace is akin to the way that judgments about respectability are used to draw symbolic boundaries in diverse urban settings. For Skeggs (1997), respectability has been central to the creation and maintenance of class hierarchies and continues to exert an influence on how people behave, speak, and make decisions regarding the individuals and groups they associate with. Skeggs' work is especially relevant given its focus on how middle‐class anxieties about social order are expressed through judgments relating to housing and domesticity. More specifically, Skeggs describes how judgments on the organization of the home, childcare practices, and control of family members have been used to identify unrespectable women and modes of femininity (1997, p. 11), while also stressing that access to the mechanisms used to generate and display respectability is mediated by articulations of class, race, gender, and sexuality.

In the context of superdiverse urban neighborhoods, questions of respectability are often posed in the context of debates about community, belonging and togetherness (Elias & Scotson, 1994). In investigating these scenarios researchers have employed the concept of symbolic boundaries, that is, “conceptual distinctions made by social actors to categorize objects, people, practices, and even time and space” (Lamont & Molnár, 2002, p. 168). As Wessendorf (2020) and Wimmer (2013) have shown, this conceptual framing—which includes a sensitivity to shifting boundaries—allows us to focus on the dynamics of exclusion *and* inclusion. This makes the approach amenable both to convivial settings, and their showcasing of “happy togetherness” and friction, and superdiversity, and its combination of inter‐ and intra‐ethnic differences and interactions.

In Newham, East London, Wessendorf (2020) uses ethnographic fieldwork to highlight the boundaries of inclusion and exclusion drawn between established ethnic minority residents and newcomers from Eastern Europe, while noting the importance of convivial practices and matrices of power. Long‐standing migrant communities claimed that new arrivals from Eastern Europe showed no willingness to “blend in,” violated codes of civility and order by drinking alcohol and begging in public, and—in the context of widespread socio‐economic precarity—capitalized on their whiteness by taking a disproportionate number of service‐sector jobs. However, these long‐standing residents also expressed empathy with newcomers based on their own parents' struggle to be accepted and an acknowledgment of the extra challenges posed by the febrile anti‐immigrant politics of the Brexit referendum and its aftermath. Indeed, a small number of social programs created forums where the established‐newcomer boundary was crossed via forms of convivial labor. For example, a program which ran at a local school offered cooking classes where parents from different ethnic backgrounds could break from their everyday patterns and create new forms of conviviality. Ultimately, though, this individual boundary crossing did not lead to any movement of symbolic boundaries, with socio‐economic precarity and structural racism continuing to foreground differences between established and newcomer groups.

Wessendorf's (2020) focus is on how established ethnic minority groups apply their own paradigms of respectability to the behavior of newcomers from Eastern Europe, rather than the distinctions drawn within Eastern European migrant groups along the lines of respectability and any cross‐racial engagement with these distinctions. Indeed, the literature on conviviality and superdiversity tells us little about the distinctions drawn within an ethnic group on its own terms. This is despite Vertovec's (2007) “diversifying of diversity” being bound up with emerging patterns of inequality, segregation, cultural mixing, and mobility which can be experienced differentially *within* ethnic groups. Charsley and Bolognani (2017) describe how the use of a particular stereotype—the “freshie”—among British Pakistanis reflects and reinscribes intra‐ethnic distinctions based on notions of difference, similarity and disgust. Through an analysis of internet comedy videos and their own qualitative data, they shed light on the way that cultural and social capital, and corresponding notions of sexuality, circulate within transnational social fields in marking the “freshie,” typically a recent Pakistani migrant, as a figure of abjection and disgust. More specifically, the “freshie” is “mocked for lack of cultural capital, suspect in terms of immigration status, ridiculous in their efforts towards social acceptance, and exhibiting dubious sexuality and disgusting bodily practices” (2017, p. 57). That said, these views were not held and applied uniformly. Education and social class allowed some migrants to transcend the boundaries associated with cultural capital, while a number of British Pakistani contributors to internet fora objected to new migrants being negatively stereotyped. Furthermore, the clarity of this stereotype contrasted with a blurring of the “us‐them” boundary in the British Pakistani community caused by trends like British Pakistanis spending periods of their childhood or young adulthood in Pakistan.

Unsurprisingly, then, intra‐ethnic boundary making is also implicated in power dynamics and situated practices. However, because Charsley and Bolognani (2017) focus on uses of the “fresh” stereotype within one ethnic group, they do not explore how these dynamics and practices may play out in cross‐racial deployments of the stereotype. Also, with the authors primarily tracking articulations of the stereotype across internet platforms, we do not get a clear impression of how it may be used in private and semi‐public space.

As noted by Tyler (2017, 2020), most studies of convivial, superdiverse urban settings analyze interactions and exchanges which take place in public and semi‐public spaces, paying little attention to the role of everyday constructions of home in the paradoxical dynamics of inclusion and exclusion, conviviality and racism. Through a number of papers which examine the dynamic of inter‐ethnic and inter‐racial relationships in a suburban town within commuting distance of London, Tyler (2015, 2017, 2020) shows how white working‐class residents (from English, Scottish and Anglo‐Italian backgrounds) exhibit attitudes to their British Asian neighbors which encompass (and entangle) racism, xenophobia and Islamophobia together with “routine and respectable expressions of interethnic conviviality” (2017, p. 1904). Here, various of dimensions of home and home space—including the material, ethereal and symbolic—are articulated with the white self in both including and excluding racial, ethnic, and national *others*. For example, white residents could cite the good “neighbourliness” of British Pakistanis evidenced through their participation in the day‐to‐day civilities and reciprocities associated with historically white neighborhoods (for example, in helping to carry shopping bags), while also endorsing stereotypical ideas about South Asian collectivism and insularity (indicated by an alleged lack of integration into the predominantly white neighborhood and nation) (2020, p. 233).

In this article we describe how white British residents of a superdiverse housing estate use inter‐racial exchanges to learn about the intra‐ethnic distinctions drawn by “respectable” British Asian neighbors and flatmates, then attempt to apply these to judgments about the “unrespectable” behavior of other British Asian residents and visitors. As well as documenting the forms of convivial labor involved, we ask how these exchanges reflect the metropolitan paradox together with notions of whiteness and invocations of home.

The convivial labor we describe below reflected a desire articulated by white British residents to avoid making inaccurate and possibly racist statements about their British Asian neighbors and visitors to the estate. As a result, they sought to learn more about the distinctions used *within* British Bangladeshi and British Pakistani communities through inter‐racial engagement. However, despite being motivated by an anti‐racist stance and an apparent desire to distance themselves from forms of extreme whiteness (Lawler, 2012), white British residents failed to appreciate key details and nuances of the intra‐ethnic stereotypes they had learned about. This led to elements of each stereotype and the labels associated with them being inaccurately applied, extending the wider discrimination faced by certain British Bangladeshi and British Pakistani residents and visitors. The use of intra‐ethnic stereotypes by white British residents also reinforced the boundaries *between* racial groups, as the people that white residents sought out for knowledge of intra‐ethnic distinctions felt uncomfortable at being identified as purveyors of “insider knowledge,” particularly where white residents went on to deploy this knowledge in an imprecise and/or inappropriate way.

## 3 SETTING AND METHODS

The paper is based on four‐years of ethnographic fieldwork conducted on an inner‐London housing estate, Lashall Green (LG).^2^ (The fieldwork was part of wide‐ranging study encompassing a range of issues such as housing biographies and informality.) LG is located in Northtown, one of the most diverse areas in the UK in terms of ethnicity. According to the 2011 census, 34% of Northtown residents were from Black, Asian, or minority ethnic groups. People from Bangladeshi backgrounds represented the largest minority ethnic group in Northtown (5.67%), followed by those from Black African backgrounds (4.90%). A further 22% were non‐British white residents including those from Irish and various European backgrounds.

The estate comprised 148 units ranging from studio‐flats to large two‐bedroom maisonettes. Of the 178 adult residents who responded to a survey, 40 (22%) identified as white British, 25 (14%) as Black African, 25 (14%) as white Irish, 22 (12%) as Bangladeshi, 13 (7%) as Kosovar, 11 (6%) as mixed white‐Black, 9 (5%) as Greek‐Cypriot, 7 (4%) as Pakistani, 7 (4%) as Afghan, 4 (2%) as Black‐Caribbean, 4 (2%) as Arab, 2 (1%) as mixed white‐Asian, 2 (1%) as white French, 2 (1%) as white Spanish, 2 (1%) as Chinese, 1 (1%) as white Italian, 1 (1%) as white Brazilian, 1 (1%) as Jewish. In line with the “diversifying of diversity” identified and explored by Vertovec (2007), there were also significant intra‐ethnic differences between residents. As a result of Margaret Thatcher's Right to Buy policy (introduced in 1980), around a third of the units had been bought by tenants (a significant proportion of which—in line with wider trends—had subsequently been sold to private landlords). The majority of private renters on the estate were either students or young professionals. This range of occupancies—social housing, private renters and owner‐occupiers—complicated the already diverse ethnic landscape of the estate. For example, differences along the lines of social class, generation, region, and religious observance separated residents belonging to the same ethnic group.

Throughout the course of the study, both authors were immersed in the social life of the estate and its environs. The first author, who is from a mixed‐race (white‐Asian) background, lived on LG for four years while working as a coach and mentor at a local school and youth club. The second author, who is from a white British background, worked for twenty years as the area's youth worker. This meant that the study's sample was skewed slightly in the direction of young people, plus residents living in the same block as the first author.

Of the 84 semi‐structured interviews carried out during the project, 23 pertained to disorder, respectability, and notions of ethnic and racial difference. However, given the specific focus of this paper, we used the findings from 15 interviews as the basis of our analysis. We do not claim that the interviewees who feature here are representative of residents of all superdiverse housing estates in urban areas and do not wish to homogenize any of the groups they belong to. It is very possible that the intra‐ethnic boundaries explored here are not reproduced in other superdiverse areas, and that there are white British people in superdiverse areas—those who grew up in these areas, for example—who engage with intra‐ethnic boundaries differently. We follow a strategy adopted in other studies of conviviality and the metropolitan paradox (Back & Sinha, 2018; Tyler, 2020) in using a handful of cases to develop a detailed analysis of how granular intra‐ethnic distinctions figure in the complex dynamics of inclusion and exclusion.

The interviews lasted between 30 and 75 min and were recorded using a digital dictation device. While the majority of the interviews took place on the estate, in interviewees' flats, shared gardens and semi‐covered communal areas, others were conducted in a local youth club. Interviews were transcribed, with transcriptions and field notes coded using NVivo to identify patterns and key themes. A series of shorter, unplanned conversations was also written up and checked with discussants for fairness and accuracy. All interviewees and discussants provided full written consent for their participation in the study.

## 4 INTRA‐ETHNIC BOUNDARY MAKING ON LASHALL GREEN: “FRESHIES” AND “TEPIS”

“Fresh off the boat,” or “freshie” in its contracted form, is a stereotype used in a number of migration contexts and transnational social fields (Charsley & Bolognani, 2017). On LG, the stereotype was used to mark intra‐ethnic boundaries, with slightly different connotations and variations across ethnic groups and among clusters of “proximate” ethnic groups (especially British Pakistanis and British Bangladeshis). However, perhaps unsurprisingly given the area's history of migration‐related superdiversity, the term was also used in a more general, cross‐racial sense, especially among young people. This usage was reminiscent of how judgments about respectability and corresponding “us‐them” or “insider‐outsider” boundaries have been applied across racial groups in other neighborhood settings (Wallman, 1982; Wimmer, 2004), though, as we will see, it could also accommodate notions of intra‐ethnic particularity. In this section we detail various applications of the term, while in the following section we describe how the stereotype, and a cognate term, “tepi,” were deployed by white British residents during (and resulting from) inter‐racial exchanges with their British Bangladeshi and British Pakistani neighbors and flatmates.

Nik, a twenty‐eight‐old whose Greek‐Cypriot grandparents had moved to Northtown in the 1960s, lived in a ground‐floor studio flat. He described how the fresh stereotype was used to mark boundaries within his peer group.

Nik: … at school there was kids coming from all over. Nobody's really from here, right? … we had new kids from Kosovo, Africa, added to classes where it's Bengali, Somali, Greek, Irish … some were fresh … But so were we!

We asked him to elaborate on what “fresh” meant in this context.

Nik: … it was about fighting. Boys that were nervous … jumpy … Some of these kids came straight from war zones, man. And they could flip. From nothing to fists, weapons … uncles! But then some of us were fresh, too. And we had been here from day (one).

Here being “fresh” is portrayed as a mindset associated with young male migrants who, because of an insecurity born of their newcomer status, would react violently if they felt challenged by other young people. Indeed, Nik's words highlight how a “civility towards diversity” (Wessendorf, 2014), conditioned by the reality that everyone was ultimately from somewhere else, could be fragile. In failing to observe rules around the acceptable bounds of conduct, young men whose families had moved from “war zones” in Africa and Eastern Europe were identified as having a distinctive potential for violence. But Nik emphasized that only some new arrivals would possess this mindset, and that other young men who had been born in Northtown could remain “fresh” in their attitudes and behaviors. His account demonstrates how the fresh stereotype had been sharpened and elaborated through convivial practices which take place in public and parochial urban settings (Wessendorf, 2014a; Wise & Noble, 2016), while supporting the assertion that, in superdiverse settings, perceptions of new migrants are shaped by individual histories of settlement and exclusion (Wessendorf, 2020).

Other young residents confirmed that while the label tended to be applied to newcomers, it could also refer to young people who had been born and raised in the area but still exhibited a “fresh” mentality. Eighteen‐year‐old Hiba, whose Somalian parents had settled on LG in the 1990s, explained her understanding of the stereotype.

Hiba: … it was mostly (applied to) boys, but not always. 'Cos girls could be violent too … mainly new kids but again not all the time. But (it) was not always the same. So another thing would be, not being aggy (aggressive), but being kind of naive and lagging behind, and this was the thing with some of the Bengali kids who have been here for (a long) time.^3^

So aside from violent potential, social and cultural capital were used to define “fresh” among young people, with British Bangladeshi youngsters (and, by extension, their families) being associated with this deficit. This sliding from general “fresh” criteria to more specific ethnic traits, hinted at by Nik and elaborated by Hiba, played out in the everyday social dynamics of the estate. If a young person known to be “fresh” in the sense of possessing violent potential passed through the estate, young residents tended to avoid engaging with them, beyond exchanging nods or glances. Conversely, a minority of young British Bangladeshi residents labeled fresh in the sense of being naïve and unknowing were treated with benevolence, though occasionally subjected to teasing and mild derision (sometimes from other British Bangladeshi youngsters). Other specific applications of the term came through in discussions about residents and their behavior, with one British Bangladeshi family being positioned outside the bounds of respectability.

The Osmans, who lived in a first‐floor maisonette, were often criticized for their noisy, disorderly behavior, with their “fresh” characteristics being used in the drawing of symbolic boundaries. Jhanvi, a twenty‐six‐year‐old British Bangladeshi teacher who lived in the same block, made reference to these characteristics.

Jhanvi: They make so much noise. Dad having loud phone calls in the middle of the night—obviously to family back home. Having dinner really late, too, obviously when he finishes work, with the kids running around. Bad English … not bothering to speak to people, Mum hardly leaves that place. It's classic fresh Bengalis, really!

Her comments reflect the way that notions of respectability center on the organization of the home, family life and childcare (Skeggs, 1997). And though the whole family is criticized here, the biggest share of responsibility is apportioned to “Dad,” minicab‐driver Abdi. While his patriarchal control of the family was itself seen as a problematic “fresh” characteristic, the fact he used this influence to orchestrate and reproduce “fresh” behavior in his wife and children compounded the problem.

The rhythm of family life was oriented around his patterns of work and a mixing of time zones. In this way, his navigation of transnational social fields was considered both unsuccessful and disruptive, while his patriarchal dominance was seen as doubly problematic given it was exercised by a man who had been born in Britain (Ahmad, 2008). Indeed, as with British Pakistani invocations of Pakistan as “backwards” in relation to Britain (Bolognani, 2014; Charsley & Bolognani, 2017), Abdi's conduct (especially with regard to family life and ongoing ties with Bangladesh) signaled a lack of social development and excessive traditionalism.

Abdi defended his behavior: “I don't think it's fair … I go to drive, I come home … I do everything I can (to limit noise). But I have just reached (returned) after work all day. So I'm tired and just want to eat.” He also commented on the decision to build close‐knit family relationships: “I come out on Saturday morning with my kids to wash the car. Otherwise I don't need to come out. We don't have so many racist(s) like we did a long time ago. But we stick together still.”

He explained the reluctance to venture out of the flat with his family by alluding to experiences of racism in the past. Indeed, the “ethnic capital” (Borjas, 1992) he amassed by “sticking together” came at the expense of “dominant” forms of social and cultural capital—which might have value in the mainstream labor market—as well “non‐dominant” types of capital (Carter, 2003)—the kind of tastes and preferences synonymous with cultural status among young British Bangladeshis. The strategy he deployed should therefore be understood in the context of exclusion from the mainstream labor market and marginalization in relation to British Bangladeshis who possess more in the way of “non‐dominant” cultural capital. In Skeggs' (1997) terms, Abdi recognized that a combination of racism and class prejudice gave him limited access to the mechanisms used to generate and display respectability.

It was difficult to identify a stereotype with one ethnic group on the estate. However, there was a term which was used almost exclusively by British Pakistani residents, though its field of application was wider than just this ethnic group. This was “tepi,” a term which had similar connotations to “freshie” but elaborated on some key vectors of intra‐ethnic distinction and connected them with uncivil, disorderly behaviors. We first encountered the term during a conversation with flatmates Sheri and Sheena, British Pakistanis who had moved to central London from Manchester and Southall, respectively, to study at a local university. They lived with fellow undergraduate, Amy, whose (white) Scottish parents had settled in the midlands, in a two‐bedroom maisonette where the living room served as a third bedroom.

Sheri: I'm tired of ignorant people at uni(versity) or on nights out asking me about Muslims and Pakistanis. All this stuff about Pakistanis being backward, being terrorist nutters and grooming young girls in Bradford or whatever. If that stuff's even true, these are people from the total backwaters of Pakistan, different rules (apply) there. They're tepis, man. Low caste, illiterate—probably not even here legally—just don't have a clue … There's a load of these boys at the uni down the road. We literally couldn't be more different.

We asked Sheri and Sheena about the origin of “tepi” and to elaborate on its meaning.

Sheri: I don't know for sure. But you hear these boys when they're round here for house parties. They're so loud and sloppy, and the way they speak. Can't shake that accent even though they're born here … I said caste (before) but I use that (word) when I'm explaining these things to other people. It's more like families and where they're from in Pakistan … plus where they live here.

Sheena: There's the religion thing, too.

Sheri: Oh yeah. These boys claim to be religious but they will go out with white or Black (non‐Muslim) girls right up until their parents sort them out (via an arranged marriage). We're not about anything being arranged.

Sheena: Yeah, totally. As we said before, we'll drink and smoke the odd zoot (joint), but that's discreet. Plus we're not claiming to be strict hijabis.

Like the “freshie” stereotype unpacked by Charsley and Bolognani (2017), “tepi” seemed to have connotations relating to language, gender, sexuality, as well as human and cultural capital, within transnational social fields. “Tepis” were identified by subtle linguistic markers, including the pronunciation of particular words and plosive sounds. These were connected with the regions and districts in which they had settled; supposedly places where British Pakistanis from “low‐status” families originating in “backward” regions of Pakistan and with dubious citizenship status stuck together and lived insular lives. The term was used exclusively in relation to men who, despite claiming to stick to religious (Islamic) codes of behavior, had relatively promiscuous sex lives before surrendering to their parents' choice of life partner. Their suspect religiosity was also reflected in their loud, brash, and “sloppy” behavior. Additionally, they possessed either no educational credentials or the “wrong” ones (despite these being awarded in the UK) (Charsley & Bolognani, 2017; Qureshi et al., 2012). As with the “fresh” stereotype applied to the Osmans, then, the “tepi” label was used to criticize someone's positioning vis‐à‐vis transnational social fields. The tepi's navigation of, and attempts to move beyond, his transnational social field were deemed inappropriate, inadequate, contradictory, disruptive and ultimately unsuccessful. His actions were marked indelibly by his low‐status background (defined geographically in both the UK and Pakistan) and its correlates in the areas of citizenship, speech, religion and educational credentials, which scuppered his attempts at integration. This was consummated by marriage to a young woman selected by his parents and his subsequent retreat into insular, parochial, and patriarchal communal life.

## 5 WHITE BRITISH RESIDENTS' USE OF INTRA‐ETHNIC BOUNDARIES

In this section we explain how two white residents learned about and deployed the symbolic boundaries bound up with the “fresh” stereotype, before doing the same for the “tepi” stereotype in the case of a white undergraduate student whose two flatmates were British Pakistani.

Ann and Terry, both in their mid‐to‐late twenties, worked as teachers at local schools. The couple had lived in a studio flat beneath Abdi and his family for a number of years since moving to Northtown from suburban locations in the south of England. They described becoming aware of the “fresh” stereotype and learning more about the characteristics attached to it through conversations at work and on the estate.

Ann: We've had issues with the Bangladeshi family upstairs. Noise … eating very late, very late phone calls.

Terry: We're not from here so didn't know much about Bangladeshi culture … But from speaking to other teachers plus other colleagues, you could learn.

Ann: … the kids will say “fresh”. We would never have used that (word) … it's helpful, because, otherwise we might have said “problem family” or, I don't know, “nuisance family”. But that's too strong anyway and just not accurate really. And the last thing we want to do is start talking about, you know, race. This way just works better all round.

Terry: Plus it's nice to feel like you're settling in and learning about the place. I mean, Jhan(vi) has helped us a lot. Not just in explaining why what's happening upstairs might be happening, but in talking with Abdi for us.

Here inter‐racial engagement is prompted by concerns over conduct that is perceived to be disorderly and unrespectable. Aware that their own attempts to capture the behavior of their British Bangladeshi neighbors—the “problem” or “nuisance” family—were inadequate, and wary of falling back on racial stereotyping, they used convivial labor to learn about a supposed intra‐ethnic distinction. Their resulting judgment was more detailed in that it alluded to intersections between ethnicity, class, and regional differences. Also, having learned about the distinction from a British Bangladeshi, in their eyes the subsequent judgment was more authentic. It was also interesting that their judgment was formulated in the context of everyday constructions of home, in both a material sense—the practical issues of sharing a boundary with other people—and a wider, more symbolic sense—where “settling” into the locale involved learning about their new, superdiverse surroundings. As in the suburban location explored by Tyler (2015, 2017, 2020), it was possible to identify connections between these notions of home and constructions of whiteness, with Ann and Terry's conscious attempts at “settling in” seemingly motivated by a desire to distance themselves from the “hyper‐whiteness” (Tyler, 2015) and “extreme whiteness” (Lawler, 2012) which the media and politicians have used to position working‐class white people (Lawler, 2012; Tyler, 2015).

That said, there were important implications resulting from Terry and Ann's deployment of the stereotype. Jhanvi explained her discussions with Terry, Ann and Abdi.

Jhanvi: Yeah I've gone down and talked with Abdi. I feel (for) them as they're right below all that. So I try to be all diplomatic and explain why the family are a little fresh. But … I'm in that weird in‐out situation. And I do feel a bit uncomfortable about them (Terry and Ann) throwing the term around … it probably helps them make sense of things, but I'm not sure they fully get it. Like, it can be awkward when they assume that because the parents are fresh the kids will be fresh in the way that the kids at school say it. I mean, those kids may miss the odd day (at school), but they aren't trouble at all.

Here Jhanvi alluded to a situation in which Terry and Ann had wrongly assumed that, as the Osman family had been labeled “fresh,” the children in the family would be prone to misbehave and even act aggressively at home and at school. This was another assertion that connected practical concerns about home life (including complaints about noisy neighbors) with navigation of public and semi‐public space, plus ongoing attempts to adapt to the wider superdiverse locale (Tyler, 2020). However, Terry and Ann's use of the term had not accounted for the different ways that the stereotype was deployed locally. Recall Hiba's statement above, where she described how “fresh” could be applied, on one hand, to aggressive and/or violent young people and, on the other, to those who lacked any aggression but were perceived as naïve and slow to adapt to their surroundings. For Jhanvi, Ann and Terry failed to appreciate these differences in usage. Their own deployment of the stereotype was inappropriate, likely to cause offence and could unfairly extend the discrimination faced by an already marginalized family.

Jhanvi was also uncomfortable about her ambiguous “in‐out” position within these exchanges. That is, in being asked by Ann and Terry to explain what “fresh” means in the context of respectability among British Asians—and, more narrowly, among the British Bangladeshi community—Jhanvi detected a presumption on her neighbors' part regarding the underlying sameness of all members of that group. As she put it: “It's really, like, ‘Tell me why that person isn't like you’. But, like, you're only asking because of where I'm from.” So while the discussion between Ann, Terry and Jhanvi centered on intra‐ethnic boundaries—with these boundaries ostensibly excluding a low‐status, “fresh” British Bangladeshi family from the bounds of respectability—more subtly it also served to foreground the racial boundary between Jhanvi and her white British neighbors.

The way that Amy, a white British undergraduate student in her early twenties, engaged with the notion of “tepis” teased out certain characteristics of the stereotype while underlining power differentials and again reinforcing the symbolic boundaries that existed both within and between groups. She commented on getting to grips with this stereotype with the help of her British Pakistani flatmates, Sheena and Sheri.

Amy: I grew up around Birmingham so I get the “fresh” thing. But this was next level. The talking thing, I'm not sure I can even hear it now. It's pretty subtle. They get frustrated with me and try to spell it out: “Ts from the tongue, not from the teeth”. But when you meet these boys it makes sense and it's like “I totally get it” … we would say “wide boy” but that's not it. These girls have it down!

As well as underlining the subtlety of the linguistic markers attached to it, Amy's comment pointed to the granularity and assumed authenticity of the “tepi” stereotype (as opposed to an inadequate and inauthentic alternative, the “wide boy”^4^). Amy also stresses her metropolitan credentials and an ongoing willingness to perform convivial labor; like Ann and Terry above, she seems eager to construct a non‐extreme, unobtrusive form of whiteness which is amenable to the superdiversity of her surroundings. However, as with Ann and Terry's use of the “fresh” stereotype, Amy's deployment of “tepi” as an intra‐ethnic distinction could make her flatmates uncomfortable. Sheri described her reasons for explaining the stereotype to Amy.

Sheri: She didn't get it. But, why would she? Like, we're all Muslim but not strict. Like, no pork but anything else, fine. But we're honest about that. And don't judge. Our parents know that really but we just never talk about it. And never put it on social media. But these boys, they will do all these things but swear to their parents they aren't doing them, and then criticise us for doing the same thing. Same as they have fun with Black girls, white girls then want a “pure” hijabi bride at the end of it. And all the while their parents think they're little angels. This is why we explain it. Plus, if you saw our houses, they're the same, almost. My parents won't borrow money so there, like with Islam, the differences aren't that obvious.

Here Sheri reiterated the key vectors of difference which underpinned the use of “tepi” as an intra‐ethnic stereotype. But she also elaborated on intersections between gender, religion, and class which mapped onto the differences in speech, region, and legal status outlined earlier in the paper. Tepis, so the stereotype seemed to run, were raised in families that were strictly religious and, being male, were granted an elevated status by their parents. Their straying from religious codes as young men was deemed dishonest, not only because their parents would not (knowingly) tolerate such behavior, but because they would criticize any young Muslim woman who did not conform strictly to the codes of Islam (and, by extension, with their ideal image of a prospective bride). This perceived dishonestly, hypocrisy and chauvinism made pointing out the corresponding vectors of intra‐ethnic difference important. For Sheri, the absence of material differences to signal her family's higher status (because of Islamic codes which discourage moneylending at interest) made this a particularly urgent task, with intra‐ethnic boundaries policed more vigilantly and “tepi” characteristics tied to intra‐ethnic vectors of difference in a more specific and strident way.

Amy's application of the stereotype did not always take account of these subtleties. This frustrated Sheri, who implied that more “frame negotiation” (Wise, 2016) was required before Amy could use the stereotype appropriately.

Sheri: Amy will mess it up. Like there'll be male friends over and after they've left she'll say, “Ooh, he's a bit tepi for you”. And I'll have to point out that he's not at all—just a Pakistani boy with an accent. You get mad and think, “Do you really just think we're all the same?”

As with Jhanvi, Sheri was concerned that misuse of the stereotype would extend the discrimination faced by British Asians (in this case, young British Pakistani men). She also questioned her true position in relation to the inter‐racial and intra‐ethnic boundaries traced during discussions with Amy about “tepis,” with Amy's occasional misuse of the term serving to highlight the racial divide between flatmates. For Amy herself, the blurring of boundaries was an additional source of confusion, with her flatmate Sheena sometimes applying the stereotype to British Bangladeshi residents of the estate.

Amy: Yeah, that messes things up. I mean, sometimes just other Pakistanis, sometimes Bengalis, too.

Sheena explained the slippage in terms of social distance and, more specifically, feeling less assured than Sheri about applying the term to British Pakistanis.

Sheena: I'm not like these boys, (our) families (are) different as well. But we're not massively different. I grew up with boys like that, but now our paths aren't the same at all. Plus, you know, this area isn't very Pakistani anyway. Just loads of Bengalis … Yeah, they're Muslim like us. (But) they don't really speak English. And just stick to their own. Not like us at all.

Here Sheena blurs and shifts boundaries in ways that partially account for Amy's confusion, with context and relational differences coming into play.

## 6 CONCLUSION

Building on recent attempts to bring together conviviality and boundary making (Wessendorf, 2020), along with insights into intra‐ethnic othering (Charsley & Bolognani, 2017), in this paper we have examined how white British residents of a superdiverse housing estate draw on the intra‐ethnic distinctions explained to them by British Bangladeshi and British Pakistani neighbors and flatmates. They used these distinctions to formulate judgments about the unrespectable behavior of other British Bangladeshi and British Pakistani residents and visitors. In doing so they underscored a number of Skeggs' (1997) assertions about the dynamics of respectability, including the centrality of organization of the home and family life to allegations of unrespectability, and differential access to the mechanisms used to produce and display respectability along various lines of social division.

The nature of the distinctions which underpinned intra‐ethnic boundaries was hardly surprising. The use of the “fresh” stereotype and its assertions regarding lack of cultural capital, excessive traditionalism and positioning within transnational social fields chimed with Charsley and Bolognani's (2017) exploration of this stereotype among British Pakistanis. The “tepi” stereotype touched on similar themes, but its use placed particular emphasis on vectors of intra‐ethnic difference which did not correspond with obvious signs of material wealth and status; for example, Sheri was eager to stress that although her family home may be smaller than those belonging to the parents of “tepi” young men, a set of differences relating to interpretation of Islamic codes, regional origin and place of settlement, gender relations and cultural capital set them apart.

In using their new‐found knowledge of intra‐ethnic boundaries to formulate judgments about British Bangladeshi and British Pakistani neighbors and visitors, the white British residents we focus on here drew on notions of home in a material and more symbolic sense. For Ann and Terry, it was the intimacy of sharing a physical boundary—with one home quite literally on top of the other—that led to concerns about unrespectable rhythms of family life. But, as they saw it, their commitment to settling and making a home in a superdiverse area entailed using convivial labor to learn about the intra‐ethnic boundaries that existed within the local British Bangladeshi community. Similarly, Amy used convivial labor to learn about the intra‐ethnic boundaries applied to British Pakistanis (and to a lesser extent, British Bangladeshis) by her flatmates Sheri and Sheena. This labor was performed with a view to understanding the conversations that took place in her flat and the judgments her flatmates made about the behavior of low‐status visitors and acquaintances.

However, despite their proud use of convivial labor to “settle in” at home, which seemed to correspond with a desire to distance themselves from extreme whiteness (Lawler, 2012)—in favor of what they hoped would be a more unobtrusive whiteness—white British residents' deployment of intra‐ethnic boundaries in the formulation of judgments about unrespectable behavior was perceived by British Asian interviewees as both offensive and exclusionary. It was not only that the reproduction of these boundaries intensified the marginalization of certain low‐status British Bangladeshis and British Pakistanis. In failing to recognize some of the nuances of the “fresh” and “tepi” stereotypes, white British residents risked extending the discrimination faced by British Asians by inaccurately applying elements of a stereotype—as in the case of Ann, Terry, and the Osmans—or inaccurately labeling people—as in the case of Amy and the young British Pakistani men who visited her flat. Furthermore, the British Asian people that white residents sought to learn from—Jhanvi, Sheri, and Sheena—felt they were asked to occupy an ambiguous “in‐out” position by virtue of being quizzed about intra‐ethnic boundaries. This positioning, combined with the inappropriate deployment of stereotypes by white British residents, served to reinforce the divide between white British and British Asian interviewees.

Our findings add to the literature on conviviality and the realities of the metropolitan paradox by showing how intra‐ethnic stereotypes are used in cross‐racial judgments about respectability. Just as Wise's (2005) interviewees spoke both positively and negatively about their Chinese neighbors during a single interview, in this paper we have seen how the same act of convivial labor—engaging with flatmates or neighbors across racial lines to learn about the intra‐ethnic stereotypes used to understand the reasons for unrespectable behavior—can express the subtle dynamics of inclusion *and* exclusion. Ostensibly these inter‐racial exchanges seemed to unite white British and British Asian residents within the bounds of respectability (through the exclusion of “unrespectable” British Bangladeshi and British Pakistani residents/visitors). However, white British residents' attempts to avoid making sweeping and potentially racist statements by borrowing from intra‐ethnic stereotypes, especially when these stereotypes were deployed imprecisely, could still convey assumptions about the underlying sameness of British Asian communities, thereby distancing and offending even those they sought to include.

## Data Availability

The data that support the findings of this study are available on request from the corresponding author. The data are not publicly available due to privacy or ethical restrictions.

## ENDNOTES

^1^
In analyzing “inter‐ethnic” and “inter‐racial” dynamics we recognize the contested nature of “race” and “ethnicity” as both terms and concepts. We follow Meer (2014) in understanding ethnicity as having a looser definition than “race.” That is, while the former centers on the definition of group membership along the lines of real or imagined features such as language, culture, religion and collective memory, the latter premises group membership on a supposed biological unity (often imputed to the group by outsiders). However, while this notion of “race” as an objective category has been discredited, with social scientists showing how it is the result of social construction, belief in “race” and racial difference persists and continues to have very real consequences. The proliferation of inter‐ and intra‐group differences in superdiverse settings makes it increasingly difficult to draw a clear line between assertions of ethnic and racial difference. As a result, our use of the terms “inter‐ethnic” and “inter‐racial” is tentative and provisional.

^2^
The names of all places and people (besides London) have been replaced by pseudonyms.

^3^
There was a slippage between the terms “Bengali” and “Bangladeshi,” with the terms used interchangeably by almost all respondents.

^4^
The Cambridge Online Dictionary defines a “wide boy” as “a man who is dishonest or who deceives people in the way he does business.”
